# Designing user interfaces for content simplification aimed at people with cognitive impairments

**DOI:** 10.1007/s10209-023-00986-z

**Published:** 2023-03-24

**Authors:** Lourdes Moreno, Helen Petrie, Paloma Martínez, Rodrigo Alarcon

**Affiliations:** 1 Computer Science Department, Universidad Carlos III de Madrid, Leganés, Spain; 2 Department of Computer Science, University of York, York, United Kingdom

**Keywords:** Cognitive accessibility, Design, People with intellectual disabilities, User interfaces

## Abstract

Cognitive accessibility aims to make content more accessible for people with cognitive impairments, such as the elderly and people with intellectual and learning disabilities. In this sense, it is possible to design an accessible user interface from a cognitive point of view. As a contribution, this article presents cognitive accessibility design patterns and their application in designing the Easier web system's user interface. The Easier web system provides a tool that assists in the understanding and readability of text content geared towards people with intellectual disabilities. It detects complex words and offers easier replacements and other resources such as a definition of the complex word. In addition to applying the design patterns, user tests with people with intellectual disabilities and older people have been carried out to evaluate the cognitive accessibility of the Easier system's interface. The results indicate that people with cognitive impairments know how to use the interfaces and have a satisfactory experience. In addition, a design proposal to provide a glossary mechanism to be used in web interfaces with simplified texts is presented and validated.

## Introduction

In addition to being a crucial way of accessing information and services, the Internet is also a fundamental tool for participating in society. Nevertheless, the current efforts being made concerning Internet accessibility [1] do not adequately support all individuals, such as those with cognitive and learning disabilities. For example, according to [2], while online shopping has become commonplace for people in general, individuals with cognitive disabilities are not comfortable purchasing items online.

Although the needs of people with specific disabilities, such as those with sensory and physical disabilities, are much better understood, the same cannot be said for the cognitive barriers in user interfaces or the complications involving the lack of understanding and readability of text content, which so far has not been studied sufficiently.

Cognitive accessibility barriers primarily affect people with intellectual, language and learning disabilities directly related to the mental processes of perception, memory and reasoning. Additionally, accessible interfaces and simplified text content can benefit other individuals, such as deaf and deaf-blind people, the elderly, the illiterate and immigrants with a different native language. According to certain studies, many people with cognitive disabilities face the same usability problems as others. The impact experienced by people with cognitive disabilities due to these issues is, however, more harmful [3–5].

Understanding texts that contain unusual words is more complicated than understanding texts with more straightforward terms, but this is not the only cognitive accessibility barrier some individuals face. Poorly designed user interfaces that, for example, provide excessive information, a failure to give headings, inappropriate line spacing and a lack of clear, step-by-step instructions, among other problems, can also lead to significant accessibility barriers [6, 7].

Several initiatives pose cognitive accessibility guidelines on user interfaces, such as the Web Content Accessibility Guidelines (WCAG) 2.1 [8], a standard that deals with cognitive accessibility in a very tangential way, and the “Cognitive and Learning Disabilities Accessibility Task Force (W3C-COGA)” initiative [7], in addition to some literature. Standards-based documentation is frequently extensive and is often, therefore, poorly applied by designers. This work proposes cognitive accessibility design patterns based on standards as a solution space. In addition, these patterns have been used to design the Easier system, the second contribution of this work. Following a user-centred design approach, the Easier system has been designed and validated with the participation of people with cognitive impairments, such as people with intellectual disabilities and the elderly.

Related work is presented in section two. Section three presents a proposal of design patterns for cognitive accessibility. Section four briefly describes the Easier system. Section five shows the design of the user interface. The user evaluation of the user interfaces is shown in section six. Finally, some conclusions and future directions are offered.

## Related work

There are specific directives that provide guidelines on how to make content more accessible for people with cognitive and learning disabilities. One of these initiatives is the Web Content Accessibility Guidelines (WCAG), a part of the W3C’s WAI and the benchmark standard used by most of the world’s regulatory frameworks [9]. When the specific guidelines included in the WCAG are followed, web content is made more accessible for people with cognitive and learning disabilities. Nevertheless, research was carried out on websites designed under the WCAG guidelines and browsed by people with cognitive disabilities [5]. The results of this usability study showed that while most participants could access the Internet, they could not use websites successfully. Problems related to user satisfaction and perceived usability were also identified, in addition to several issues regarding web browsing. This study clearly demonstrated that the WCAG guidelines do not adequately address the needs of individuals with cognitive disabilities.

For this reason, the “Cognitive Accessibility at W3C” [10] initiative was created. It includes a range of resources, such as related guidelines and informative guide that go beyond the standards, as well as ongoing work in cognitive accessibility support at W3C. Among these guidelines, the “Cognitive and Learning Disabilities Accessibility Task Force (W3C-COGA)” is worth mentioning and must be considered. One of this task force’s main goals is to guide how to make websites and user-friendly applications for users with cognitive impairments, focusing on both the design process and the designs themselves. More research is needed to understand precisely the effect these cognitive disabilities have on the use of web-based media and resources.

Another key initiative is the Easy-to-Read guidelines that focus on people with intellectual disabilities and other affected groups. These guidelines address some aspects of text presentation and include pictograms, symbols and other augmentative elements as a glossary, as well as focusing on text comprehension and readability. These guidelines have been disseminated thanks to the work of the International Federation of Library Associations and Institutions (IFLA) [11] and their publication of the document “Directives for Easy-to-Read Materials”. Furthermore, the document “Make it Simple: European Guidelines for the Production of Easy-to-Read Information” was published by Inclusion Europe (formerly the International League of Societies for Persons with Mental Handicap (ILSMH)) [12]. These two documents constitute the beginning and de facto standards for easy-to-read content.

Another important initiative is the Plain Language initiative, geared more towards the general public [13, 14]. This initiative aims to promote the simple language used in electronic governmental content and information, as well as to improve the quality of the customer service provided to all citizens [15]. Governments are currently providing accessible information on their eAdministration and eHealth platforms, among others, and, therefore, have been creating guides and adapting many of their public communications. Certain similarities can be seen among the initiatives established in these guidelines, but there are still some significant differences [16]. In addition, it is essential to combine these guidelines with cultural aspects according to the audience to which it is directed [17].

Regarding the interaction of people with disabilities with the content and services offered by the Internet, several problems, such as cognitive accessibility issues that occur when the back button does not work correctly, were discovered. Individuals who have difficulty reading will find this action more complicated [4]. Simulation results are presented in the study [18] demonstrating how a slight decrease in how well cues like link labels are interpreted will often result in a substantial increase in the time dedicated to searching a website. This indicates that issues with searches which cause relatively significant problems for good readers may cause catastrophic problems for poor readers. Some studies have emphasized interaction problems: [19, 20] show that text entry can be problematic, [21] indicate that users have difficulties in recognizing hyperlinks, and demonstrate that typing and reading instructions are often actions that are difficult to perform [5].

Certain studies deal with the difficulties of people with intellectual disabilities when interacting with the Web. Results from [4] indicated barriers such as difficulty entering query text into search engine boxes and in the browser bars, difficulty selecting from a large amount of text, and difficulty getting something out of the text due to impairments in reading ability. In [22] provided recommendations for online training, such as the addition of navigation indicators and contextual aids, the cleaning up of screen pages both graphically and textually, and the predominant use of video-based content. Work described in [23] established web content guidelines concerning various elements such as text size, browsing consistency and page design, icon use, images, written text, style, margins, links, line spacing, and screen layout. Some studies on web accessibility for people with cognitive disabilities analysed various aspects of accessibility and usability of different websites [5]. It was found that the ability to navigate was affected by unclear and inconsistent navigation, non-standard interaction techniques, lack of click-ability, user's willingness to scroll through pages, and user's ability/willingness to read instructions. The work [24] advocates adaptive design because the pattern of specific cognitive deficits when browsing the Internet differs for each individual. The authors indicate that it is necessary to work on the user profile and adapt the interface based on these profiles.

Natural Language Processing (NLP) methods can be employed to improve accessibility to text content, for instance, lexical simplification, as is described in this work. There have been some works, such as [23, 25, 26], which deal with the simplification of texts for certain groups of individuals with disabilities by using methods that have been utilized to provide accessible content for people with autism. Moreover, some works involve lexical simplification to avoid using complicated vocabulary because people with aphasia could find it difficult to understand [27, 28]. Likewise, the study [29] seeks to simplify texts for people with dyslexia. The Easier system, [30] offers automatic text simplification in Spanish for individuals with cognitive disabilities. The added value of the Easier system is to provide, in addition to lexical simplification, information such as synonyms, definitions and pictograms within an accessible interface oriented towards people with disabilities.

## Design patterns

Accessibility guidelines concerning the use of simple language and grammatical structures in texts are included in the WCAG guidelines and the W3C-COGA documentation. Additionally, these also offer interface design objectives, including suggestions for both their components and interactions that make cognitive processes simpler for people and reduce the need to rely on memory, thus promoting cognitive accessibility.

All documentation mentioned above has been analysed in this work, resulting in the proposal of a set of Design Patterns that can assist in designing web pages and applications (see Table 1). The heuristic evaluation process utilized to create web interfaces optimized for cognitive accessibility may also be beneficial.

**Table 1:** Proposal of cognitive accessibility design patterns

COD	DESCRIPTION	HOW IS THIS ACCOMPLISHED	COGA
1	Clear title that summarizes the purpose of a webpage’s content	Is a clear heading used in the upper part of each page that describes its purpose?	4.2.1
2	Make the purpose of each section clear	Does the web document have a logical structure? Are clear headings provided that briefly define the purpose of each section?	4.3.1- 4.3.3
3	Clearly describe the process of the steps	Are completed steps, the current step, and pending steps clearly indicated in a multi-step process?	4.2.4—4.3.1
4	Presentation features are used to group the different content blocks	Are presentation and graphic elements used to group and highlight information?	4.2.6—4.3.3- 4.4.10
5	Mini videos	Is video and audio content grouped into a series of short, well-labelled mini-videos and presented in a logical order that can be navigated? Is a summary provided?	4.3.5- 4.4.8
6	Make clear which elements are controls and how they should be used	Are control elements clearly identified? Are button elements clearly identified?	4.2.5—4.2.6
7	Each region and its controls can be clearly recognized	Are clear dividers used to distinguish the different content sections on a webpage? Are interactive elements, such as scroll bars and buttons, close to the content they can affect?	4.3.3—4.4.10
8	Logical order is followed for the submenu items, which can be easily identified	Is it easy to detect what submenu items there are and how to access them? Are the actions needed to open the submenu items easy? Is the visual hierarchy of the submenus easy for the user to understand?	4.3.2
9	Consistent visual design and common design patterns are used	Are common design patterns used that are familiar to most users? Is a consistent visual design used among groups of web pages?	4.2.2 -4.2.3
10	Symbols are used to help the user	Are there symbols, images and pictographs that help users understand? Do the images convey a unique meaning?	4.2.7 -
11	The most important content on the page can be found easily	Is the important content visible without the need to scroll down the page? Is there a search function provided?	4.3.4 -4.3.6
12	Common words are used	Are familiar and clear words used, and are they very frequent? Are unnecessary words avoided?	4.4.1
13	A simple tense is used in written language	Is an easy-to-understand tense used, such as the present tense or active voice?	4.4.2
14	Do not use double negatives or clauses within clauses	Is a simple sentence structure used that does not include double negatives to express a positive? Are clauses within clauses that can be confusing avoided?	4.4.3
15	Literal language is used	Is the literal language used and refers to concrete things?	4.4.4 -4.4.12
16	In instructions, each instruction is separated	When instructions are given, is each step separately, and is it supported by the use of lists?	4.4.5 -4.4.9
17	Be precise and concise	Are short blocks of text used with short paragraphs? Is one single topic or idea conveyed per paragraph?	4.4.5
18	Clear punctuation is used in texts	Is correct punctuation used in texts to improve readability and comprehension?	4.4.6
19	A summary of long documents is provided	Is a summary of long documents provided? Does the abstract use common words and short sentences, and is it written in an easy-to-understand style and tense?	4.4.8
20	Simple alternatives to mathematical content are provided	Are simple alternatives provided for numbers and numeric concepts?	4.4.13
21	Designed to prevent people from making mistakes	Does the form design reduce the possibility of the user making a mistake? Are suggestions for corrections provided?	4.5.4
22	An option to undo errors made is provided	Is the user allowed to check their work and correct any errors? Once the user has fixed an error, can they easily go back without having to redo additional steps?	4.5.2—4.5.5
23	Clear labels and instructions are used	Are instructions provided at the beginning of the process? Are clear error messages delivered?	4.5.6—4.5.7
24	Form inputs are flexible	Are variations in user inputs, such as currency, time zone and location, accepted?	4.5.8
25	Wait times with data loss are avoided	Are wait times avoided, and, if there are any, is the user informed about the time required to complete the process?	4.5.9
26	Status information is provided	In each process step, is the user informed about its status and whether or not the task has been completed successfully?	4.5.10
27	The user is identified and informed when content is changed	Are changes to context, functionality, configuration, path, and orientation initiated only at the user's request or are there readily available mechanisms to disable such changes?	4.5.3—4.9.1—4.5.1
28	Interruptions are avoided	Are interruptions avoided? If they cannot be avoided, is it easy to control changes to the content?	4.6.1
29	An excessive amount of content is avoided on the page	Are users provided with only five options or fewer on each screen? Is there any unnecessary content?	4.6.2 – 4.6.3
30	Login can be accomplished without relying on a good memory	Can users log in and register without using cognitive skills?	4.7.1—4.7.2—4.7.3
31	Users cannot be trusted to memorize information	Is the information required at each step provided to allow a user to continue in a sequential process?	4.7.5
32	Help is provided for understanding complex tasks and information	Is content provided that helps users understand complex information and successfully complete the tasks described without requiring further external assistance?	4.6.4—4.8.2—4.8.3
33	Help is provided for handling non-standard forms and controls	Is help provided on complex forms when there are multiple steps, unusual interactions, non-standard controls, or required fields that do not support auto-completion?	4.8.4
34	Human support is provided	Is a person who can provide help and support easily accessible? Access to human assistance should never require the user to manage complex menu systems, such as voice menus with different options	4.8.1
35	Reminders for events are provided	Can the user set a reminder for the date and time of events? For example, once an appointment is established, does the user have the option of adding it to their calendar automatically?	4.8.7
36	Finding different types of help and comments is made easy	Are mechanisms provided that allow the user to request help or report problems?	4.8.5—4.8.6
**37**	Contrast	Is sufficient contrast provided between the foreground and background content? Do you ensure that the background does not obscure the foreground content?	4.4.11
38	Advanced features	Are customization and adaptation functionalities provided? Is a simplification service provided?	4.9.2.—4.9.3 -4.9.4 -4.7.4

Table 1 shows the set of design patterns organized as follows: The first column shows the code of the pattern, the second column includes a brief description, the third column offers questions to support the design and evaluation of each pattern, and finally, the fourth column indicates which objectives of the W3C-COGA it corresponds to.

## The easier system

The Easier service-oriented system^1^,^2^ aims to improve cognitive accessibility by providing a variety of resources in Spanish, promoting access to easily understandable, plain and straightforward content for individuals with cognitive impairments, the elderly and, generally, for all people.

Methods from the Artificial Intelligence and NLP disciplines are used in conjunction with Human–Computer Interaction to create accessible interfaces with simplified texts. The Easier system uses Machine Learning and NLP techniques, in addition to Easy Reading and Plain Language resources to determine if a Spanish word in a text is complex or simple. The user types a Spanish text into a text box. The Easier system then detects complex words in the text, highlights them, and when the user hovers over them, a tooltip with a simple replacement or synonym is presented. In addition, different resources are offered for each complex word to help the user improve their reading and comprehension. The system provides synonyms, a definition, and a pictogram for each complex word depending on the context of the word that appears. These resources are provided as a Glossary facility geared towards people with intellectual disabilities.

The machine learning model is trained using a range of collections of texts annotated by experts, in addition to a corpus^3^ created specifically for and by this research work by Spanish Easy Reading and Plain Language experts. In order to generate and select appropriate synonyms, dictionaries, semantic similarity techniques, and paraphrase resources have been used. Furthermore, the BERT (Bidirectional Encoder Representations from Transformers) word embedding model was used to carry out a disambiguation process and provide the most appropriate definitions of complex words within their specific context\* MERGEFORMAT [31].

The definitions provided for the words are taken from Easy Reading and the Royal Academy of Spanish (RAE) dictionaries.^4^ The “Diccionario Fácil (Easy Dictionary)”^5^ was used to provide Easy Reading definitions, while the resources provided by ARASAAC^6^ are used to offer pictograms.

The regulatory framework and the WCAG 2.1 were followed when designing its accessible user interface. Moreover, the Design Patterns presented in Sect. 5 were used to optimize the system’s design for cognitive accessibility.

A responsive design has been used for the Easier system’s web interface, making it accessible by desktop computers and mobile devices. Furthermore, there are extensions for Chrome and Mozilla browsers that users can use to identify complex words and provide synonyms for texts from any web page.

**Fig. 1:**
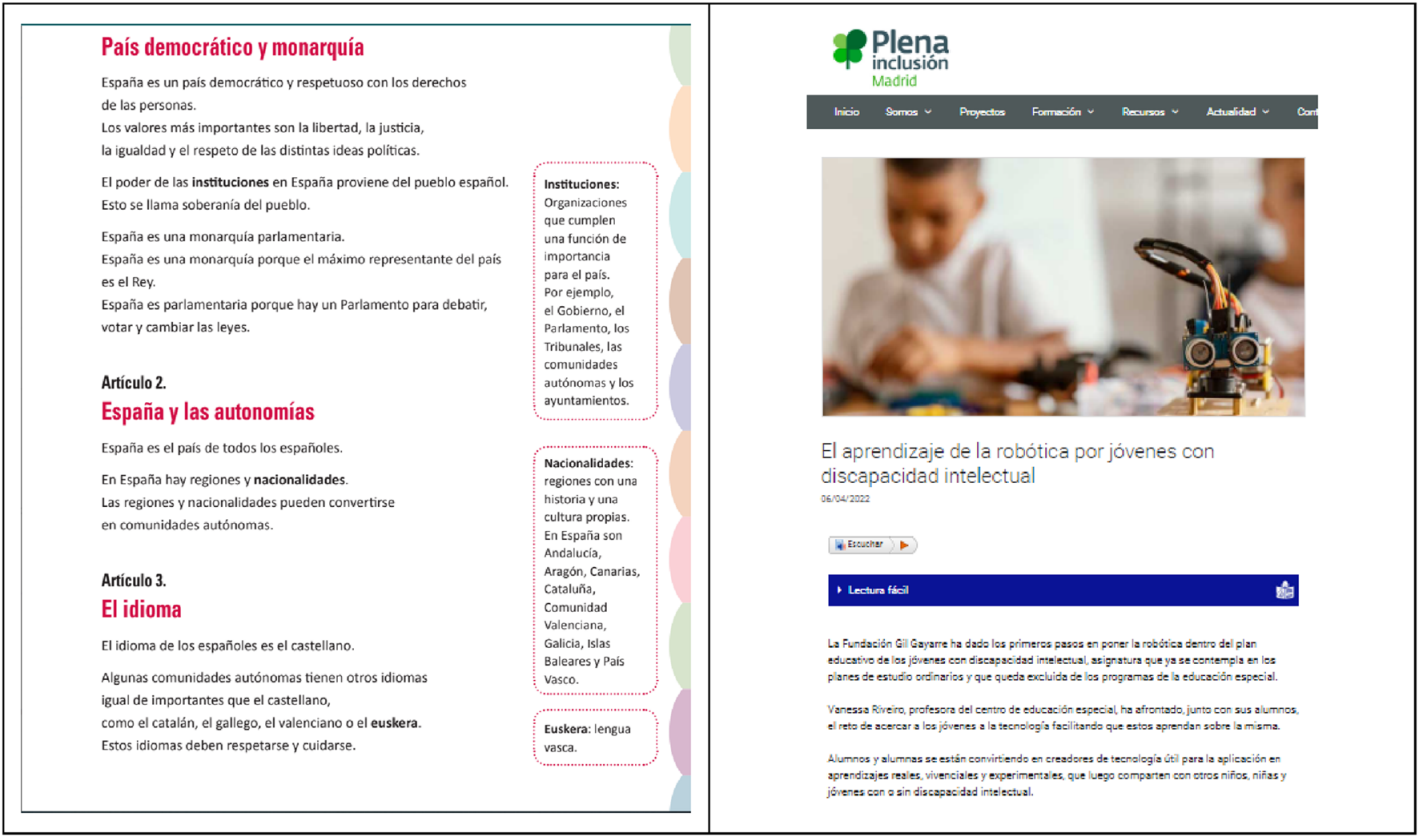
Screenshots of how the glossary mechanism is presented in (a) easy-to-read documents and (b) web pages

With regard to Glossary mechanisms, in easy-to-read texts in documents or books aimed at people with intellectual disabilities, a Glossary mechanism which explains less common or complex words through contextualization is included and is only supported by an explanation of meaning. Most references indicate that the complex word should be highlighted or put in bold, although some say the word can be underlined. It is then explained in the margin of the page as a Glossary entry (See Fig. 1a). Currently, this format has not been transferred to a web format, nor has any other related work been found. However, while it can greatly help people with disabilities, the text is presented only in easy-to-read formats on web pages without the Glossary mechanism (see Fig. 1b). With this motivation and following the accessibility guidelines of the WCAG, the Easier system interface provides a solution proposal for the design of a Glossary mechanism that includes synonyms, pictograms and definition of the complex word.

## Designing the EASIER user interface

Cognitive accessibility and simplicity were the requirements for the interface of this tool. This was achieved by employing clear content and making each step of the simplification process as understandable as possible, including all necessary instructions in plain language. All this was placed in a cognitively accessible web interface, applying the Design Patterns proposal in the design process for this purpose.

Two main web pages are the bases of the Easier system. These pages are as follows:

- Home Page (see Fig. 3): this page provides texts describing the system’s purpose and instructions on how to use it. There is only a single box in which the text to be lexically simplified can be entered. Furthermore, there is one button that executes the simplification process;
- Results Page (see Fig. 5): an explanatory text that has been adapted into plain language is included on this web page. There are two parts to the page, with one box on the left and another on the right. The entered text appears in the left-hand box with the complex words highlighted. When the user interacts with each highlighted work, help content, such as synonyms, a pictogram, and a definition, appear in the box on the right. The definition can be provided in Easy Reading language, and the Glossary mechanism is supported.

**Fig. 2:**
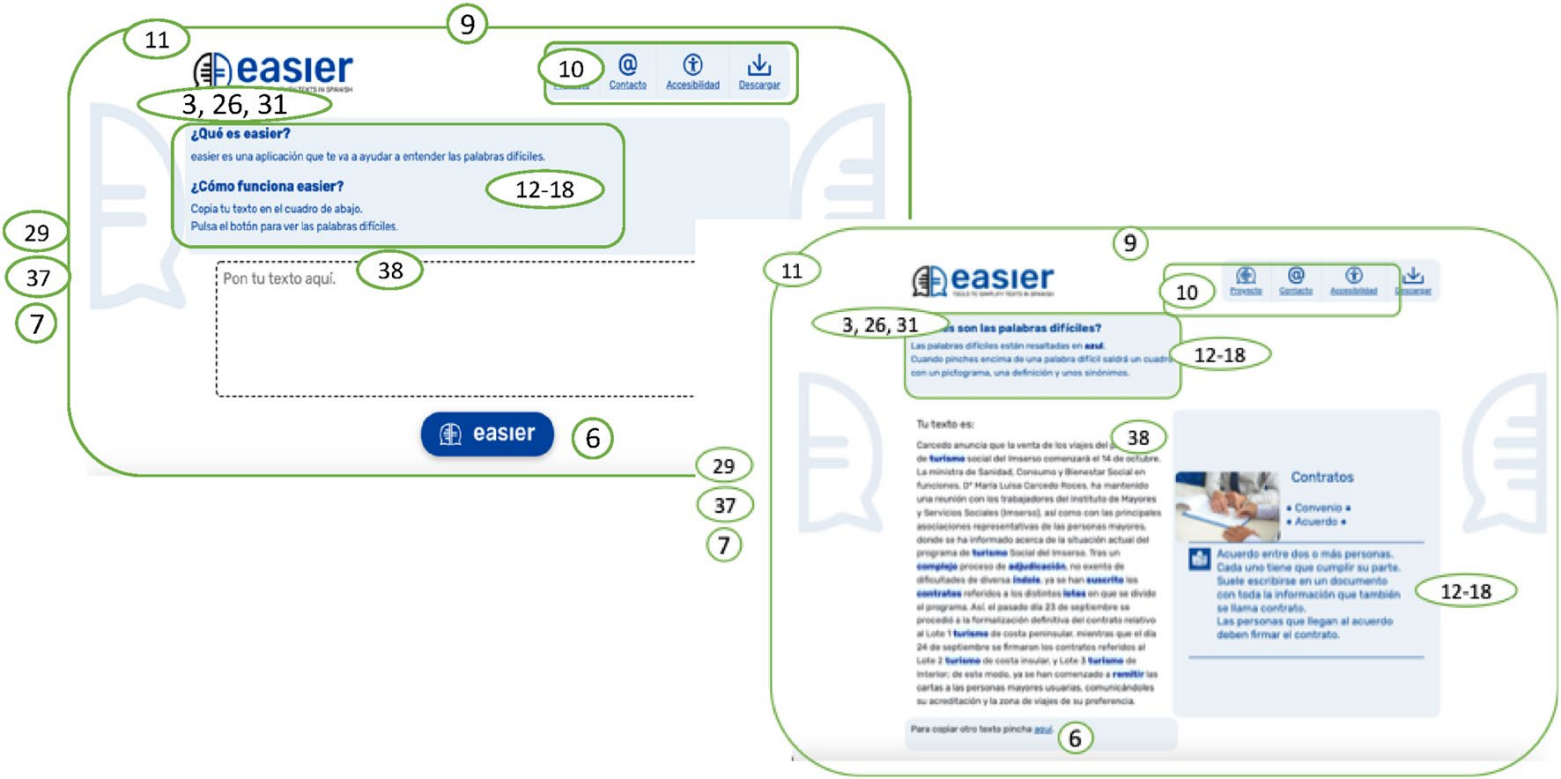
Design of the web user interfaces of the Easier system

Figure 2 shows the Easier System interface indicating the main design pattern code applied to each section. The main patterns are introduced below. Design Pattern (9) was used, defining a consistent visual design for the entire application so that the design is familiar to users. In this way, they are able to perceive that they are still in the application and that there have been no context changes.

Design Patterns (3) (26), aimed at clearly describing the process of the steps in carrying out a simplification task, were applied with this intention. In this sense, instructions were included in each step of the simplification task; these instructions were provided in plain language by an expert in plain language following Design Patterns (12) to (18). This aspect also favours reduced mental effort by not assuming users can memorize information (Design Pattern (31)).

One important Design Pattern is (11), which indicates that the most important content is visible without the need to scroll down the page. In this sense, this pattern has been followed on the home page. Additionally, no unnecessary content is included on the home page (Pattern (29)).

The button on the home page was designed in such a way as to be clearly identified and within its region (Pattern 6). Each region and its controls can be plainly recognized since clear dividers distinguish the different content sections on the web page as indicated in Design Pattern (7).

Since symbols, images and pictographs can help the user, pictograms and graphic elements are offered following the Design Pattern (10).

The WCAG has been followed and applied in accordance with WCAG 2.1 (AA level), including Design Pattern (26) concerning the colour contrast between foreground and background content.

Finally, an advanced feature is included by providing a simplification service following Design Pattern (37).

Including people with cognitive difficulties, needs, and demands in the design process is essential. Automated accessibility tests mainly focus on technical issues. While these tests are fundamental, they do not assess whether people with cognitive difficulties can successfully use the content. For this reason, it is necessary to carry out user tests with people with cognitive difficulties so that any barriers that may exist can be identified. In this sense, an exploratory user evaluation was carried out to validate the design decisions and is presented in section 6.

## Evaluation

In this section, the evaluation of the Easier web interface is presented. The research questions used were the following:

RQ1. Can people with cognitive disabilities access and know how to use the Easier application with a satisfactory experience?

RQ2. Is the proposal for the design of the Glossary mechanism in a web format that is proposed in the Easier interface, a possible and satisfactory solution?

In order to confirm these research questions, a range of evaluations was carried out with users following a user-centred design approach. First, user tests were carried out with 6 people with cognitive impairments. With the user feedback received from this process, some aspects of the interface were modified, and a usability evaluation was subsequently done with 16 people with cognitive impairments. Finally, a survey was carried out among Easy Reading professionals to complete the validation of the Glossary mechanism design proposal, which users had already validated.

### 6.1.User testing

Six individuals with intellectual disabilities and older people participated in an exploratory study based on user tests to analyse the application's usability and determine if any cognitive accessibility barriers existed \* MERGEFORMAT [32]. Participants from the Association of People with Intellectual Disabilities AMAS Group participated in the experimental sessions at their facilities.^7^

#### Participants

The AMAS Group recruited the participants. This study included the participation of six individuals (see Table 2). There were three adults with intellectual disabilities and three older people between the ages of 65 and 75. The individuals with intellectual disabilities had a mild level of disability on a three-level scale (mild, medium, advanced). There were four women and two men.

**Table 2:** Participant characteristics

USER	GENDER	Disability
U1	Male	older adult
U2	Female	older adult, low vision
U3	Female	older adult, low vision
U4	Female	mild intellectual disability
U5	Male	mild intellectual disability
U6	Female	mild intellectual disability

#### Stimuli

The two main pages of the Easier system’s web interface were used as the stimuli. With design variations in some of their elements, these web pages were evaluated by completing a task and various user tests.

#### Procedure and method

This experimental study involving individuals with intellectual disabilities was approved and supervised by an ethics committee from the Universidad Carlos III de Madrid. The various entities involved followed all COVID protocols during the entire study. Participants were informed of the purpose of the experiment, and what it entailed, and they then signed a consent form. Permission was obtained from the legal guardians of individuals with intellectual disabilities. Furthermore, a professional member of the AMAS Group, who likewise signed a consent form, carried out these tests.

All participants were required to complete a defined and simple task. They needed to interact with web pages as well as answer specific questions running the task. A professional educator worked in conjunction with the study’s lead researcher to draft the questions.

The task consisted of entering a text on the home page and then clicking on the button that would take participants to the results page that included the simplified text. The user tests can be found in Table 3.

**Table 3:** User tests of the task

TEST 1: On the home page, do you understand what you have to do on the home page?	
The purpose is to verify if the first step of the text (enter the text and click on the button) is understood (see Fig. 4)	
	Question (Q) 1.1 Do the users understand the introductory texts and explanation? Q1.2 Do the users enter or understand that they must enter the text? Q1.3 Do the users know they must click on the button once they have entered the text? Q1.4 Have the users offered any recommendations?
TEST 2: On the home page, which button is identified better? Do you know that you must interact with the button?	
The purpose is to verify which button is more easily identifiable for the users (see Figs. 5a and 5b). In other words, which button do the users believe looks more like a button and do they know they must click on it?	
	Q2.1. Which button do the users prefer? Q2.2. Have the users offered any suggestions? What type of button do they like most
TEST 3: On the results page (see Fig. 6), do the users know what they must do in the box on the left containing the results?	
The purpose is to verify if the users understand the explanation and know how to interact with the page elements. In other words, do they know they must click on the highlighted complex words? Do they know that once they click on a complex word, resources such as synonyms, pictograms or photographs, and definitions will be shown in the box on the right? Finally, do they know that a tooltip appears with a simpler synonym when hovering over complex words with the mouse?	
	Q3.1. Do the users understand the explanation? Q3.2. Do the users then click on the highlighted words? Q3.3. When the users click on the highlighted words, do they realize that the resources (synonyms, pictogram or photos, and definition) are shown on the right? Q3.4. Are users satisfied when they hover over the complex word with the mouse? Does this synonym help you understand? Q3.5. Have the users offered any suggestions?
TEST 4: On the results page, are the resources of the Glossary mechanism (synonyms, pictogram or photograph and definition) understandable in the box on the right of the web page that contains the results?	
The purpose is to verify if the users know these are the resources for the highlighted word they have clicked on and that they are able to identify what the word is, what the corresponding pictogram or photograph is, or what the appropriate synonyms are. Additionally, the purpose is to verify if the users identify the definition	
	Q4.1. Do the users understand that the following resources appear on the right-hand side of the page: complex word, synonyms, pictogram or photograph, and RAE or Easy Reading definition? Q4.2. Do the users like how the box on the right containing these resources has been designed? Which layout of the elements do the users prefer (see Figs. 7(a) and 7(b))? Q4.3 Have the users offered any suggestions regarding the layout of the various elements?
TEST 5 Do the users like the web pages' design, look and feel?	
The purpose is to verify if the users like the general design, look, and feel of the pages or if there is some element they dislike	
	Q5.1. Do the users like the design of the web page? Q5.2. Do the users like the icons? Q5.3. Have the users offered any suggestions with regard to the design?
TEST 6 Is the application useful?	
The purpose is to verify if users are satisfied with the application and to collect their opinions on whether these resources are helpful	
	Q6.1. Do the users feel or notice, that the application is useful or helpful for them? Q6.2. Have the users offered any suggestions?

#### Results

**Fig. 3:**
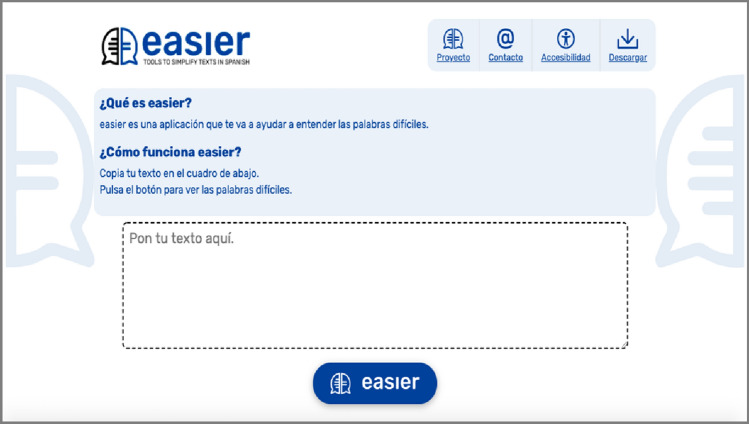
Screenshot of the Easier system’s home page

Concerning Test 1 on the home page, a certain level of confusion was experienced with regard to Q1.1 regarding whether the participants understood the explanatory texts found on the home page (see Figure 3) or not. The root of this confusion was the fact that the project’s name (“Easier”) was included in the text. Nevertheless, the users understood all page’s other explanatory and introductory texts. That being said, the visual impairments of two of the users ((U2) (U3)) caused them difficulties in seeing the texts.

With regard to Q1.2 about whether or not the users entered or understood that they needed to enter the text, all participants understood that the text must be entered in the appropriate box. All participants correctly responded and indicated the corresponding box when asked to do so.

All participants understood that they must click on the button (Q1.3). Nevertheless, the appropriate button was only correctly identified by three of the participants ((U2) (U3) (U4)). The three other participants found it difficult to complete the task ((U1) (U5) (U6)). This task was complicated for (U5) because he thought the button looked more like an image and not a button. (U1) did not identify the button correctly. (U6) identified the button correctly on her second attempt.

One of the suggestions offered by the participants (Q1.4) was that the button should be more clearly marked. (U1) made this suggestion and proposed including the explanatory text “Click the blue button” located at the beginning of the button itself. Changing the type of button used was suggested by another participant. No other suggestions were offered by any of the remaining participants.

**Fig. 4:**
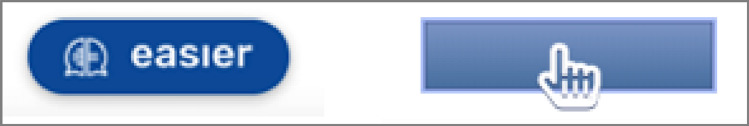
Button types **a** left button **b** right button

The participants were asked which type of button they preferred in the last question of Test 2. Two different options were given (see Figs. 4a, b). With regard to Q2.3, option (b) was chosen by four of the six participants.

**Fig. 5:**
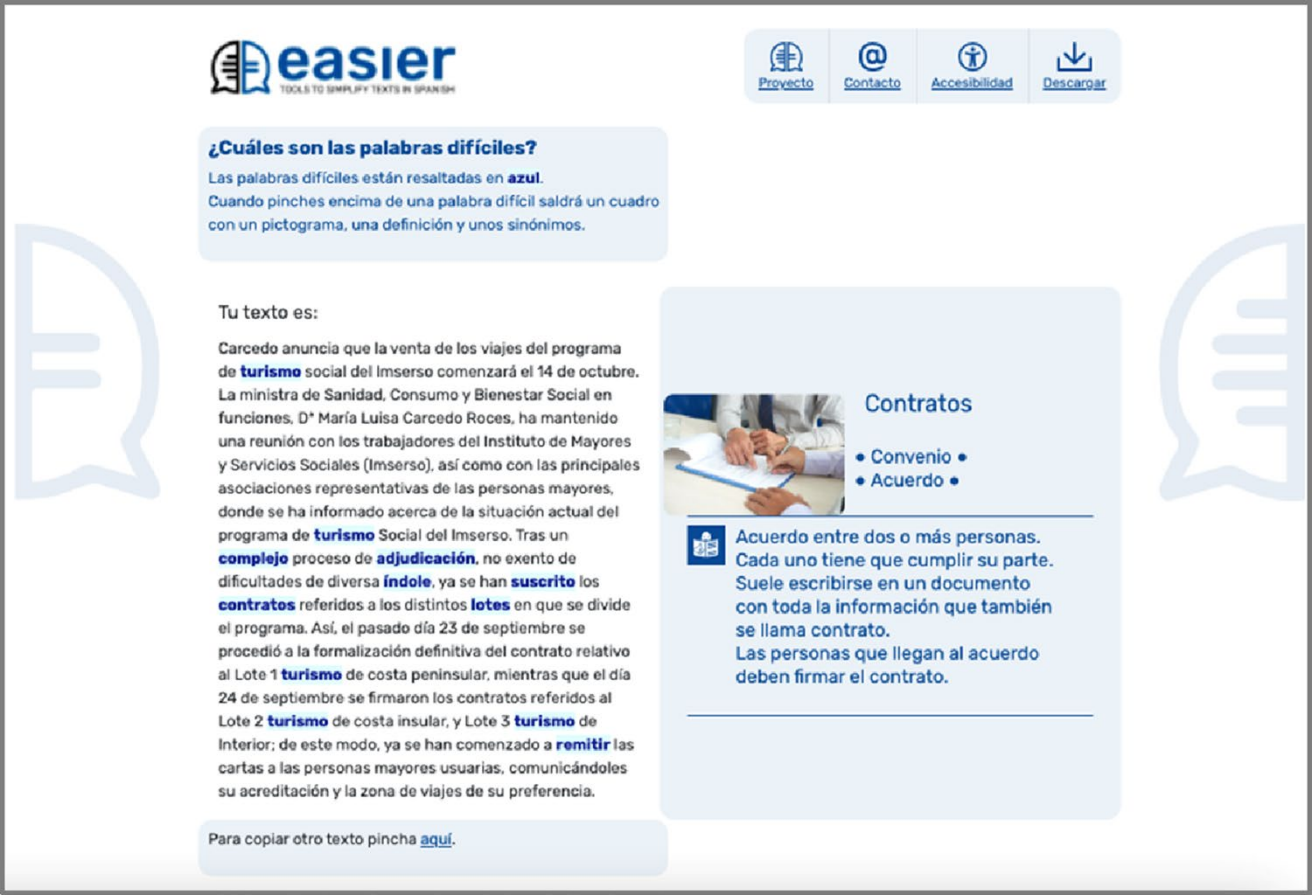
Screenshot of the Easier system’s results page

The participants were taken to the results page and Test 3 after entering the text and clicking on the appropriate button. Only one participant found it difficult to understand the explanatory text (see Fig. 5) (Q3.1), mainly because of her visual impairments.

In order to identify potential interaction barriers, participants were asked if they understood they needed to interact with the results and precisely what they were required to do (Q3.2). All of the participants were able to understand the instructions and click on the highlighted words.

Q3.3 refers to whether or not the participants understood that a pictogram or image, synonyms, and a definition would appear in the right-hand box when they clicked on a highlighted word. None of the participants had any difficulties realizing these elements appeared in a box on the right side of the page. The fact that recognizable content that helped them to better understand a text appeared in the box delighted the participants. Those participants with intellectual disabilities expressed happiness as they interacted with the system.

The participants were, overall, satisfied with the tooltip providing the simplest synonym for the complex word when they hovered over it with the mouse. Although participants (U2) and (U3) were somewhat confused by the element and its purpose, the other participants found it helpful.

**Fig. 6:**
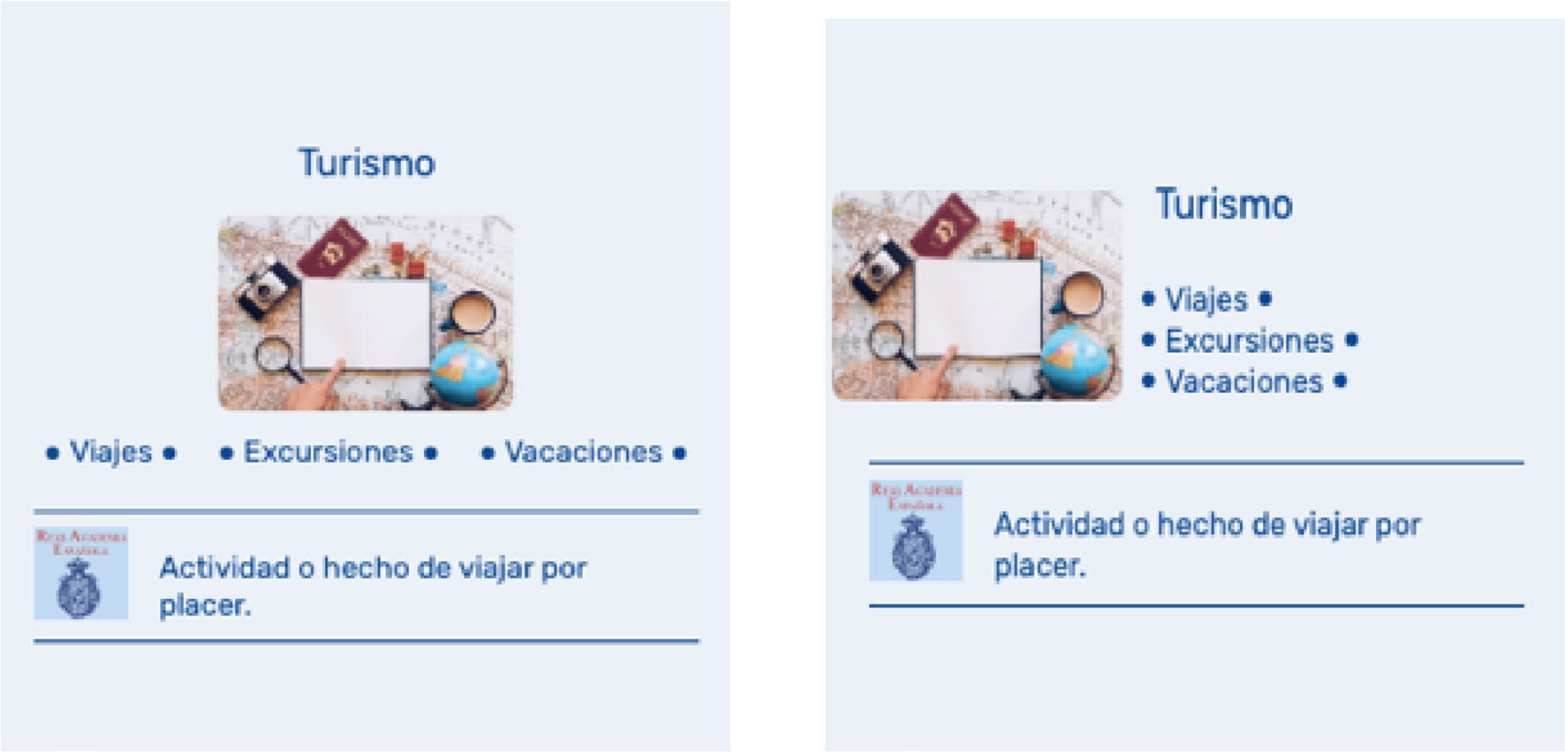
Screenshot of the right side of the results page. **a** Left box and **b** correct box

The box located on the right side of the results page (see Figure 6) was the focus of Test 4. Three of the six participants ((U1) (U2) (U3)) found it difficult to identify the various parts of the right-hand box when asked if they understood that resources such as synonyms, pictograms or photographs, and the RAE or Easy Reading definitions would appear when the highlighted words were interacted with (Q4.1). All three of these participants belonged to the older group and could not identify the various elements (pictogram, synonym, or definition). For individuals with intellectual disabilities, even though these elements assisted them in better understanding the text, what each element was did not interest them.

Participants were presented with two alternative layouts for the resources (see Figures 6a, b) to discover whether they liked the design of the right-hand box with the Glossary mechanism (Q4.2). When the elements were placed from the top to bottom, the users thought that they were ordered more logically. Design 5(a) was preferred by (U1) (U3) (U4) (U6), and design 5(b) was favoured by (U2) (U5).

With regard to Q5.1, the participants were asked if they liked the web page design. None of the participants disliked the design and thought it was clear and easy to use. Four of the participants ((U1) (U3) (U5) (U6)) found it challenging to understand the icons located in the top menu because they were not able to comprehend what they actually represented (Q5.2) fully. The icons were liked by the two other users ((U2) (U4)).

Participants were asked if they thought the application was useful and helpful in Q6.1 of Test 6. All of the users believed that this application would help them understand complex texts more easily and be useful for many people.

#### Discussion and redesign

None of the web interfaces presented any significant cognitive accessibility barriers, proving the design patterns' effectiveness. All users involved in the tests could complete the tasks and successfully interact with the application. However, specific difficulties in accessing and using the interfaces were detected, in addition to specific user preferences.

As with any group of people with some type of disability, the groups themselves are very heterogeneous; decisions are made based on the majority. The following modifications are proposed:

- Use a more distinguishable button and the solution selected by users. (see Fig. 4b)
- Use the box layout on the right (Glossary mechanism) selected by users. (see Fig. 6b)

Due to project requirements, some modifications, such as removing the name of the project (“Easier”) and icons, could not be carried out.

**Fig. 7:**
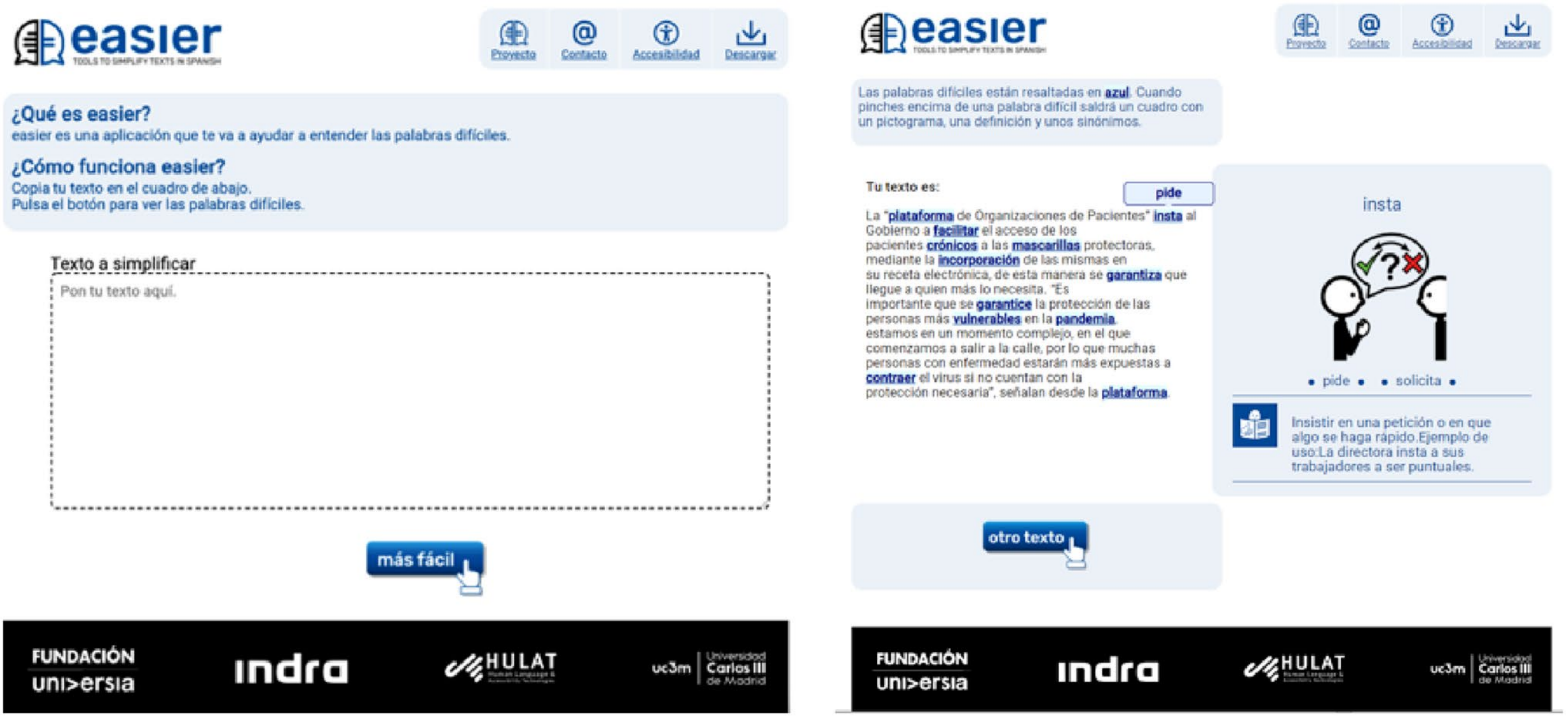
Screenshot of the **a** Home page and **b** results page

These recommendations were implemented, giving rise to the new interface shown in Figure 7. In addition to these modifications, the WCAG was followed to better identify the controls in a unique way and not only by sensory characteristics. The links were also designed in such a way that complex words are underlined as well as highlighted.

With the results of questions Q1, Q2, Q5 and Q6, the first research question (RQ1) is affirmatively supported since users knew how to use the web interfaces and had a satisfactory experience. On the other hand, they liked the results of the simplification process, the elements offered for each complex word detected, and the different resources found in the Glossary mechanism seemed useful to them. A more satisfactory design proposal was selected for them. With the latter, RQ2 can be affirmatively supported.

### Usability evaluation

After applying the design patterns and the modifications resulting from the user tests, a new version of the Easier system web interfaces was achieved (see Figure 7). Given the close link between usability and cognitive accessibility, a study with users with cognitive impairments focused on the system's usability.

#### Participants

For this experiment, the AMAS group recruited another group of participants^8^ A total of 16 participants were recruited for this experimental study.

Table 4 summarizes the demographic information of the participants, who were divided into two groups. Group 1 included 7 older people (44%) while group 2comprisedf 9 people with intellectual disabilities (56%). There was a higher concentration of men, with 10 participants (63%) than women, with 6 participants (37%), in both groups. Additionally, questions were asked to quantify the users’ experience on the Web. The majority indicated that they had more than 7 years of experience (81%), 2 (13%) participants indicated that they had between 4 and 6 years of experience. Only one (6%) participant indicated that they had between 1 and 3 years. When asked about the level of web experience, the majority considered themselves beginners with 7 (44%) participants, followed by 7 (38%) participants who considered themselves to have an intermediate level. 2 (13%) participants felt they had an advanced level, and, lastly, one (6%) user considered himself to have an expert level. In addition, the participants’ browsing frequency was asked. They were concentrated into 2 categories, 12 (75%) participants who used the Web daily and 4 (25%) participants who used the Web weekly.

**Table 4:** Participant characteristics

Features	Group 1		Group 2		All Participants	
	*N* = 7	%	*N* = 9	%	*N* = 16	%
Age						
71 +	3	(43)	–	–	3	(19)
62–70	3	(43)	–	–	3	(19)
31–61	1	(14)	5	(56)	6	(38)
30 or younger	–	–	4	(44)	4	(25)
Gender						
Male	5	(71)	5	(56)	10	(63)
Female	2	(29)	4	(44)	6	(37)
Web Experience (time)						
7 + years	6	(86)	7	(78)	13	(81)
4–6 years	–	–	2	(22)	2	(13)
1–3 years	1	(14)	–	–	1	(6)
Web experience						
Expert	–	–	1	(11)	1	(6)
Advanced	–	–	2	(22)	2	(13)
Intermediate	4	(57)	2	(22)	6	(38)
Beginner	3	(43)	4	(44)	7	(44)
Browsing frequency						
Daily	5	(71)	7	(78)	12	(75)
Weekly	2	(29)	2	(22)	4	(25)

#### Stimuli

The stimuli proposed were the web interfaces of the Easier system.

#### Procedure and method

As in the previous experiment, this study was supervised by an ethics committee from the Universidad Carlos III de Madrid, and all COVID protocols were followed during the entire study by the various entities involved. Participants were informed of the study and asked to sign a consent form. Next, they were asked to complete a simple demographic questionnaire. Afterwards, each participant was asked to complete the task. Finally, an interview following a survey created by a professional educator from the AMAS group and the researcher was done to evaluate the usability of the task.

A simple task was defined for all users to complete. During the task, users were asked to interact with the web pages and answer the questions in the questionnaire. The task was to enter a text on the home page and click on the buttons leading to the different sections of the home page. In addition, users had to interact with the contents of this web page.

#### Instrument

Accessibility and usability overlap in cognitive aspects, which is why usability is closely linked to cognitive accessibility or accessibility aimed at people with cognitive impairments and intellectual disabilities. Therefore, our initial intention was to evaluate the application with the system usability scale\* MERGEFORMAT [33]. This is an instrument commonly used in the usability testing of ICT products. Although the method to be followed includes typically an interview in which the professional educator and the researcher participate, this questionnaire had to be adapted for plain language (see Table 5) by an easy-to-read expert. Consequently, obtaining the metric associated with this instrument was impossible, so results will be given in descriptive statistics. Thus, the measures in this experiment are based on a Likert scale (1–7) supported by a SUS usability scale adapted to plain language.

**Table 5:** SUS questionnaire in Plain Language" aimed at people with cognitive impairments

Contesta al cuestionario marcando la puntuación que creas conveniente del 1 al 7, donde 1 es estar muy en desacuerdo y 7 es estar muy de acuerdo (Answer the survey, indicating a score from 1 to 7. 1 is strongly disagree and 7 is strongly agree)								
		Muy en desacuerdo (Strongly disagree)			Muy de acuerdo (Strongly agree)			
		1	2	3	4	5	6	7
1	Fue fácil usar esta aplicación (This app was easy to use))							
2	Fui capaz de completar la tarea rápido con esta aplicación (I finished the task fast with this app)							
3	Fue fácil aprender a utilizar la aplicación (I learnt to use the app fast)							
4	La información que nos da la aplicación para utilizarla es clara (The app offers clear instructions about its use)							
5	Es fácil encontrar en la aplicación la información que necesito para su uso (I can find the information about the use of the app in a simple way)							
6	La aplicación parece sencilla a simple vista (The app looks simple at first sight)							
7	Me gustó utilizar la aplicación (I liked using the app)							
8	La aplicación es muy completa (The app is very complete)							
9	En general, estuve muy contento con la aplicación (In general, I was happy using the app)							
10	Creo que me gustaría utilizar a menudo esta aplicación (I would like to use this app often)							
11	Pienso que puedo utilizar la aplicación sin ayuda ( I could use the app without help)							
12	Las funciones de la aplicación eran fáciles de usar (The app’s features were easy to use)							
13	La aplicación mantiene su apariencia y su estilo cuando cambio de un lugar a otro de la aplicación (The app keeps its appearance and style when I switch from one place to another in the app)							
14	Creo que la mayoría de las personas aprenderán a manejar la aplicación muy rápido (I think that most people will learn to use the app fast)							
15	Hay que pensar poco para manejar la aplicación (I have to think a little when I use the app)							
16	Me sentí muy seguro al manejar la aplicación (I felt very sure using the app)							

#### Results and discussion

Generally speaking, the results were very satisfactory for the participants, as shown in Table 6, obtaining a mean of 6.29 in Group 1 (older people) and 6.33 in Group 2 (people with disabilities).

**Table 6:** Usability questionnaire results (Likert Scale (1–7)) by users

Group 1				Group 2			
User	Mean (1–7)		Std	User	Mean (1–7)		Std
U1	6.00		0.94	U8	6.63		0.48
U2	5.94		0.66	U9	6.81		0.39
U3	6.00		0.61	U10	6.19		1.24
U4	6.13		1.11	U11	6.50		1.22
U5	6.81		0.39	U12	7.00		0.00
U6	6.50		0.61	U13	4.19		1.42
U7	6.69		0.98	U14	6.88		0.33
				U15	5.81		1.18
				U16	6.94		0.24
Overall Score							
Mean (1–7)		Std		Mean (8–16)		Std	
6.29		0.76		6.33		0.72	
Group 1 and Group 2							
	Mean (U1-U16)			Std			
	6,31			0,74			

Considering the results obtained for each questionnaire statement (see Table 7), all answers received good scores, with an average of 6.31. The question with the lowest mean, despite having a good value (a mean of 5.63), was question 11 (I could use the app without help), which may mean that although the application’s interfaces are usable and straightforward, for some users it may present certain difficulty with regard to autonomy. Disabled users (U11), (U12), and (U16) gave scores of 3 and 2 on this statement.

**Table 7:** Usability questionnaire results (Likert Scale (1–7)) by questions

Question	Mean (1–7)	Std	Question	Mean (1–7)	Std
1	6.31	1.10	9	6.44	0.61
2	6.56	0.79	10	6.25	1.30
3	6.25	1.48	11	5.63	1.76
4	6.63	0.70	12	6.25	1.15
5	6.19	0.95	13	6.56	0.70
6	6.13	1.17	14	6.25	1.09
7	6.56	0.61	15	6.06	0.90
8	6.50	0.87	16	6.44	0.86
Overall Score					
Mean (1–16)			Std		
6.31			1		

**Fig. 8:**
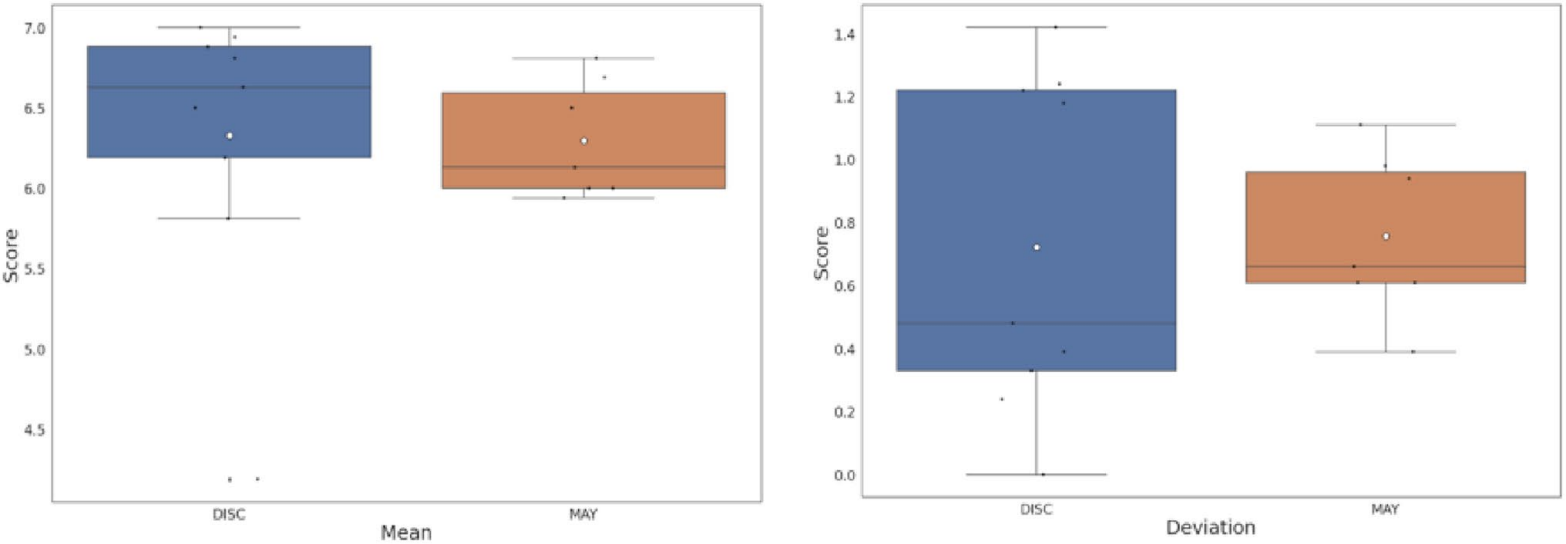
Boxplot graphs for comparative analysis of group 1 and group 2

Finally, as seen in the boxplot graphs in Fig. 8, somewhat better results were obtained in the group of people with disabilities, although, on the other hand, they present greater dispersion. In summary, this evaluation positively supports both research questions.

### Expert survey

To gather opinions on how to present the "Glossary" mechanism on web pages, an online survey was conducted by experts to prepare easy-to-read texts on what they thought of the design proposed and chosen by users.

#### Participants

Participants were recruited through a network of easy-to-read experts from different organizations to provide and evaluate easy-to-read texts, documents, and books. There were 10 participants who were only asked about their years of experience providing and validating easy-to-read texts. The values considered were as follows: (1) 0 years, (2) from 1 to 4 years, (3) from 5 to 10 years, and (4) more than 10 years. The results are shown in Table 8. As can be seen, most of them have extensive experience.

**Table 8:** Participant characteristics

User	Experience providing	Experience validating
U1	2	2
U2	3	1
U3	3	3
U4	3	1
U5	3	2
U6	3	1
U7	3	3
U8	1	2
U9	1	2
U10	4	1

#### Stimuli

**Fig. 9:**
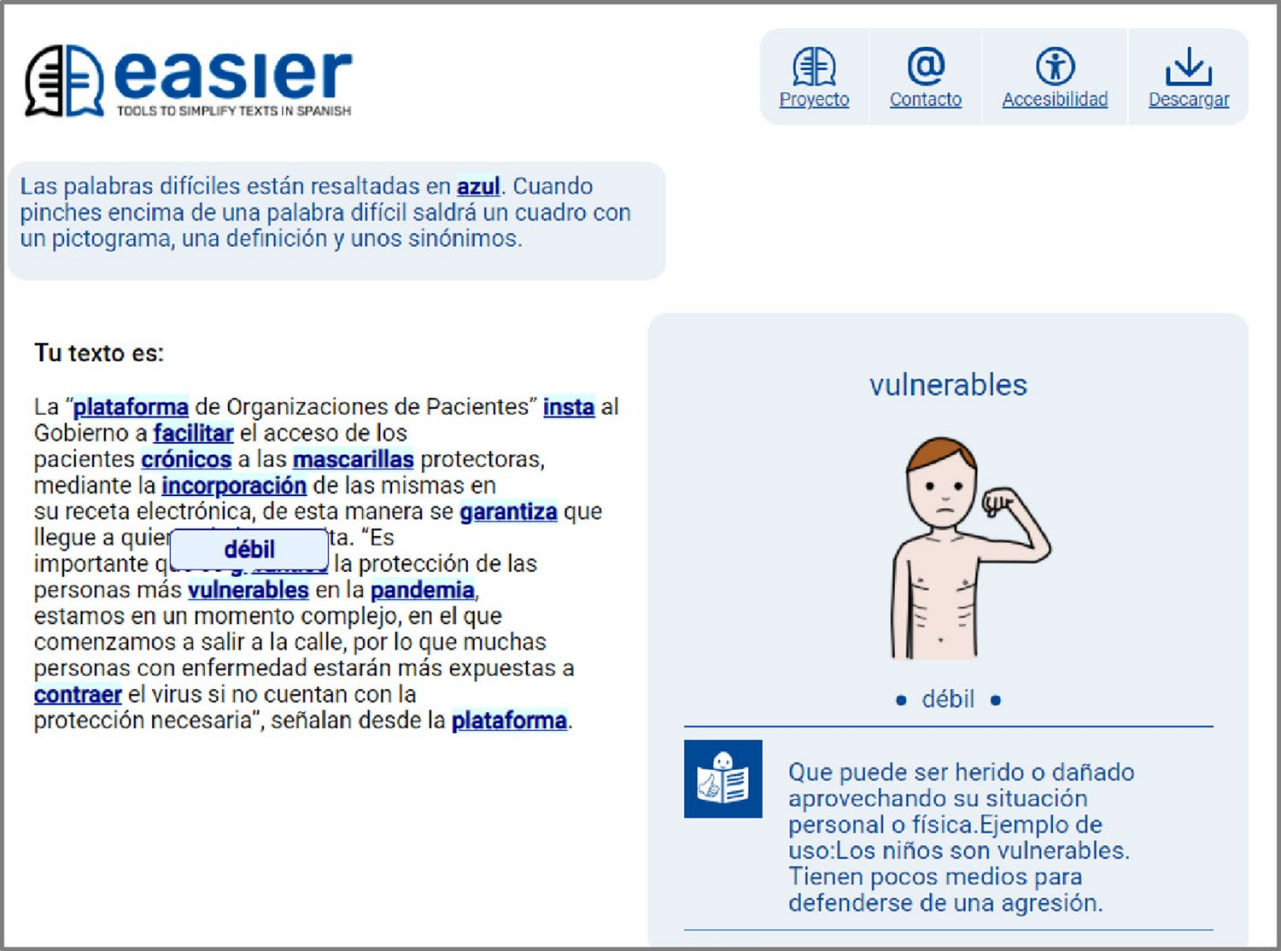
Screenshot of the web interface with a glossary mechanism in web format

The stimuli provided were a screenshot (see Figure 9) and the URL of the Easier system so that the participants could analyse the design of the Glossary mechanism.

#### Procedure and method

The method used was an online survey with the authorization of the participants. Three questions were asked. Two were related to the design of the Glossary mechanism in Easier, and another was used to gather opinions on how it could be done according to their expert view (see Table 9).

**Table 9:** Online-survey questions

Questions		Scale
Q1	As shown in Fig. 9, the complex words that need a Glossary are highlighted in the text. When interacting with each, the following appears (a) synonym by moving the mouse over it, (b) box on the right with the pictogram information, synonyms and definition How satisfactory do you think the Glossary design is?	Likert scale (1–5)
Q2	Concerning the design of the Glossary mechanism of the Easier platform. Include any comments and observations you consider relevant	Open-ended question
Q3	Can you think of a design for this context (A Glossary mechanism provided in Easy Reading texts) on web pages? How would it be?	Open-ended question

#### Results

**Fig. 10:**
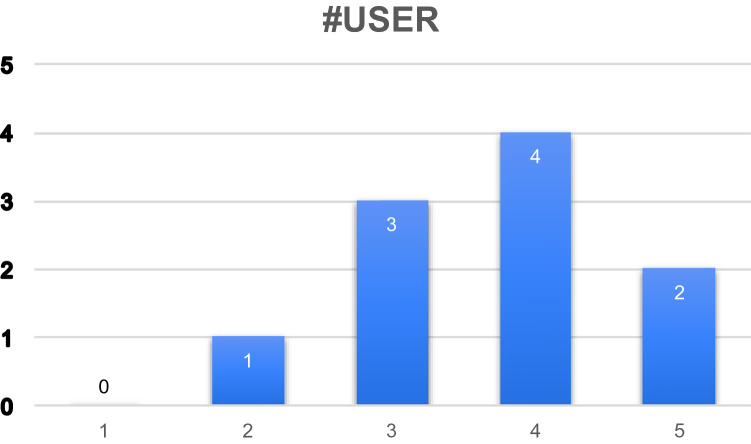
Column chart indicating the number of users by the level of satisfaction

In relation to the first question, the response was measured on a Likert Scale (1–5), where 1 was “I strongly disagree with the design used" and 5 “I strongly agree with the design used”. As can be seen in Fig. 10, the results were satisfactory: 67% (6 participants) indicated that they agreed or strongly agreed with the design; 22% ((U3) (U9) (U10)) expressed a neutral opinion and 11% (U5) somewhat agreed. No user (0%) strongly disagreed.

Concerning Q2, the participants generally liked the Glossary design. They expressed positive opinions such as "I think it is an original Glossary design" or "that includes a pictogram or image and an example of use to reinforce the definition makes that word very clear". However, some participants made comments disagreeing with some aspects of the design. (U3) (U7) felt that "I think that a synonym appears when hovering over it is not very successful since it can create confusion, and if there are many complex words, it can make reading difficult". However, another participant indicated the opposite and stated that the method used to highlight the word and the fact that when you put the cursor over it, the box with the definition appears is perfect.

One participant (U10) did not feel it was necessary to underline the words and that being in blue or putting them in bold is sufficient to distinguish complex words. This last opinion reflects the participant’s desire to directly transfer the design of a digital document or book to web pages without considering interaction mechanisms that web pages have, such as underlining to indicate a hyperlink.

Another user (U4) indicated that each resource offered should be clearly named, with labels such as Synonym, Definition, and Pictogram. Regarding the latter, the older people agreed with this participant; however, people with intellectual disabilities preferred it without the Synonym, Definition, and Pictogram labels.

Regarding Q3 (Can you think of a design for Glossary in web pages? How would it be?). There are different recommendations such as:

- The method used to highlight the word and the box with the definition appears when you put the cursor over it.
- Place the Glossary (only definition) to the right of the text at the same height as the word to describe.

In conclusion, the design used for the Glossary mechanism in Easier is a possible solution based on the answers of the participants with disabilities and the experts supporting the research question positively (RQ2). However, this issue should be explored since, currently, the easy-to-read texts on Web pages do not include a glossary when it is a reading aid resource for people with disabilities.

## Conclusions

People with cognitive impairments, such as people with intellectual disabilities and the elderly, should have full access to the Internet and its content. However, various cognitive barriers exist, and progress must be made to support the methods of presentation, interaction and the information provided. It is essential to provide accessible user interfaces from a cognitive point of view. Therefore, a proposal for cognitively accessible design patterns based on standards for web user interfaces is presented as the main contribution of this work. Furthermore, these design patterns have been used to design the web interfaces of the Easier system. The Easier system offers a web tool to help text comprehension by providing lexical simplification using Artificial Intelligence methods in an accessible user interface aimed at people with cognitive disabilities. This work addresses design aspects to achieve cognitive accessibility in the user interface.

In addition to the design patterns, different evaluations were carried out to validate the design of the web interfaces of the system with people with cognitive difficulties to identify the barriers that might exist. In addition, an evaluation was carried out with experts in easy reading. The results have shown a high level of satisfaction, giving affirmative answers to the defined research questions. The users knew how to manage the system interfaces and had a satisfactory experience. They also considered the tool to be useful. Likewise, users and experts alike considered the Easier system proposal as a possible design solution for the Glossary mechanism aimed at people with intellectual disabilities.

Finally, these positive results in the cognitive accessibility of the Easier system demonstrate the adequacy of the defined design patterns. However, they should be explored more in different user interfaces with more interactive elements.

As further work, research is being carried out on validating the design patterns through studies with people with disabilities. These studies are being done with user interfaces on everyday tasks such as making health appointments and buying transport tickets.

## Footnotes

1 http://easier.hulat.uc3m.es/.

2 https://github.com/LURMORENO/easier.

3 https://github.com/LURMORENO/EASIER_CORPUS.

4 https://www.rae.es/.

5 http://www.diccionariofacil.org/.

6 www.arasaac.org/developers/api.

7 https://www.grupoamas.org/.

8 grupoamas.org/.
